# Service use and costs in adolescents with pain and suicidality: a cross-sectional study

**DOI:** 10.1016/j.eclinm.2022.101778

**Published:** 2022-12-13

**Authors:** Verena Hinze, Tamsin Ford, Bergljot Gjelsvik, Sarah Byford, Andrea Cipriani, Jesus Montero-Marin, Poushali Ganguli

**Affiliations:** a Department of Psychiatry, University of Oxford, Oxford, UK; b Oxford Precision Psychiatry Lab, NIHR Oxford Health Biomedical Research Centre, Oxford, UK; c Department of Psychiatry, University of Cambridge, Hershel Smith Building, Robinson Way, Cambridge Biomedical Campus, Cambridge CB2 0SZ, UK; d Department of Psychology, University of Oslo, Oslo, Norway; e King’s College London, King’s Health Economics, Institute of Psychiatry, Psychology and Neuroscience, De Crespigny Park, London, UK; f Oxford Health NHS Foundation Trust, Warneford Hospital, Oxford, UK; g Teaching, Research & Innovation Unit, Parc Sanitari Sant Joan de Déu, Sant Boi de Llobregat, Spain

**Keywords:** Adolescents, Pain, Self-harm, Service use, Suicidality

## Abstract

**Background:**

Persistent/recurrent pain for more than three months and suicidality (suicide and self-harm related thoughts and behaviours) are serious and co-occurring health problems in adolescence, underscoring the need for targeted support. However, little is known about service use and costs in adolescents with pain-suicidality comorbidity, compared to those with either problem alone. This study aimed to shed light on service use and costs in adolescents with pain and/or suicidality, and the role of individual and school characteristics.

**Methods:**

We analysed cross-sectional, pre-intervention data from a large cluster randomised controlled trial, collected between 2017 and 2019 on a representative sample of 8072 adolescents (55% female; aged 11–15 years; 76% white) in 84 schools in the UK. We explored service use settings, covering health, social, educational settings, and medication for mental health problems over three months. Data were analysed using descriptive statistics and two-part hurdle models to obtain odds ratios (ORs) and incident rate ratios (IRRs).

**Findings:**

9% of adolescents reported comorbidity between pain and suicidality, 11% only suicidality, 13% only pain, and 66% neither pain nor suicidality. Approximately 55% of adolescents used services, especially general practitioner visits, outpatient appointments for injuries and contacts with a school nurse or pharmacist. Compared to adolescents with neither pain nor suicidality: (i) adolescents with pain (OR 3.79, 95% CI 2.63–5.48), suicidality (1.68, 1.12–2.51), and pain-suicidality comorbidity (2.35, 1.26–4.41) were more likely to use services and (ii) if services were used, they were more likely to have higher total costs (Pain: IRR 1.25, 95% CI 1.11–1.42; Suicidality: 1.27, 1.11–1.46; Comorbidity: 1.57, 1.34–1.85).

**Interpretation:**

In our study, adolescents with pain and suicidality reported increased contact with health, social, and educational services, which could provide an opportunity for suicide prevention. Given the diversity of identified settings, multi-sector suicide prevention strategies are paramount.

**Funding:**

10.13039/100010269Wellcome Trust [WT104908/Z/14/Z; WT107496/Z/15/Z]; 10.13039/501100008334Stiftung Oskar-Helene-Heim.

**Research in context**

**Evidence before this study**

We searched Web of Science, PubMed, CINAHL, and PsycInfo on 26th July 2022, for publications reporting on service use and costs in adolescents with pain or suicidality. We identified search terms across five categories: 1. pain (pain OR headache∗ OR migraine∗ (title)), 2. suicidality (suicide∗ OR non-suicid∗ OR nonsuicid∗ OR self-injur∗ OR self-harm∗ OR self-destruct∗ OR parasuicid∗ (title)), 3. adolescence (adolescen∗ OR child∗ OR “young adult∗” OR “young person” OR “young people” OR pediatric OR paediatric OR teen∗ OR youth∗ OR schoolboy∗ OR schoolgirl∗ OR girl∗ OR boy∗ OR kid∗ (title)), 4. service use (“service use” OR “service-use” OR “use of services” OR “resource use” OR “resource-use” OR “use of resources” OR “healthcare” OR “health care” OR “health services” OR “help seeking” OR “help-seeking” OR “seeking help” (title)), and 5. service costs (cost∗ (title)). We identified 25 references. After deduplication, ten studies were reviewed in full. Four studies were excluded that focussed on service use following treatment (n = 3) or with no useable data (n = 1). The remaining six studies focussed on service use and costs associated with primary, secondary, or tertiary care in young people i) with specific pain conditions (n = 2), ii) with vs without chronic pain (n = 2), or iii) with suicidality (n = 2). All were conducted in the United States or Canada, and it remains unclear how these findings translate to the United Kingdom. Results highlight the limited evidence on service use and costs in young people with pain, suicidality, or pain-suicidality comorbidity.

**Added value of this study**

This study describes service use and total service costs, across hospital, community, and school-based settings, in adolescents with pain and/or suicidality in the United Kingdom. The focus on a broad range of services and settings, a nationally representative sample, and distinct groups of adolescents with pain and/or suicidality closes key gaps in the literature. High data completeness, robust statistical analyses, and data recency are additional strengths of this study that together increase confidence in the generalisability of our findings. By linking service use data to the respective unit costs, this study provides a UK-based benchmark of service costs for future studies, focussing on adolescents with pain and/or suicidality. The identification of individual and school characteristics associated with service costs reveals which groups of adolescents are more or less likely to use services and to have higher costs, which has policy implications by underscoring the need for service optimisations and targeted support.

**Implications of all the available evidence**

Our findings underscore the importance of comorbidity between pain and suicidality, particularly in adolescent girls and for adolescents with higher levels of depression, anxiety, peer problems, and prescription pain medication. Adolescents with both pain and suicidality were more likely to use services and to have higher costs. This increased contact may offer an important opportunity for suicide prevention. Yet, the lack of clear dominance of one setting underscores the need for multi-sector suicide prevention strategies, focussing on young people with persistent/recurrent pain in hospital (outpatient services), community (GP services), and school settings (school nurse).

## Introduction

Persistent or recurrent physical pain for more than 3 months is a major health concern in early adolescence.^1^^,^^2^ It is often experienced in multiple locations, and in one study as many as 18% of adolescents in England, especially girls (boys: 13%; girls 23%), reported multisite persistent or recurrent pain over the past 6 months.^1^ For some, pain may persist into adulthood,^3^ with headache disorder ranking as the second leading cause of disability in 10- to 24-year-olds worldwide.^4^ Pain is associated with psychiatric comorbidities, including depression and anxiety,^5^ and at its worst suicidality, even in the absence of depression.^6^^,^^7^ The term ‘Suicidality’ will be used as an umbrella term, referring to the full spectrum of suicidal risk, ranging from thoughts about suicide and self-harm to the enactment of these thoughts. Thoughts and acts of self-harm, defined as intentional self-injury or self-poisoning irrespective of suicidal intent,^8^ are also a major concern in adolescence (10–30%).^9^^,^^10^ As self-harm in adolescence is a key risk factor of future death by suicide,^11^ the need for timely and targeted support is paramount.

Further to the distress and impairment related to both pain and suicidality, the economic impact has been estimated to be substantial (pain: £3840 million annually in the United Kingdom [UK] and $19.5 billion in the United States [US]^12^^,^^13^; hospital-managed self-harm: £162 million annually in the UK^14^). By considering overall economic costs, each service type is weighted by a respective unit cost,^15^ such that service costs can be compared within and across settings. An enhanced understanding of service use and costs is crucial to identify who accesses services and where targeted support should be delivered.

The iceberg model of self-harm postulates that self-harm in the community is much more common, yet largely hidden, compared to hospital-managed self-harm or death by suicide.^16^ Emerging research on self-harm in community samples of adolescents supports the theory that professional services are rarely contacted, even imminently before or after an episode of self-harm.^10^^,^^17^ Hence, it is crucial to study related service use in the community across diverse settings to identify key sources of support and potentially vulnerable groups who may not seek help.

Research in adults suggests that service use for mental health problems increases in the presence (vs absence) of physical comorbidities, including pain.^18^^,^^19^ Yet, knowledge on service use and associated costs in adolescents with pain and/or suicidality remains sparse, and the role of individual (e.g., age, gender, ethnicity, depression, anxiety, and peer problems) and broader contextual characteristics (e.g., school-area resource deprivation or economic deprivation) remains unclear. It is vital to learn which subgroups of young people are more or less likely to be in contact with services, and the specific services they use, to ensure that evidence-based care reaches those most in need.

This study aimed to describe service use across diverse settings and associated total service costs among adolescents with pain and/or suicidality in the UK. We tested three hypotheses:

1. Compared to adolescents with neither pain nor suicidality (reference), total service costs would be greater in adolescents with suicidality but no pain, pain but no suicidality, or pain-suicidality comorbidity.
2. Total service costs would be highest in adolescents with pain-suicidality comorbidity (reference), compared to adolescents with suicidality but no pain, or pain but no suicidality.
3. Group status (pain and/or suicidality vs neither pain nor suicidality) would be associated with using services and having higher total costs.

Furthermore, we explored whether the relationships between group status and service use and costs were moderated by individual (age, gender, ethnicity, depression, anxiety, and peer problems) and school characteristics (school-area deprivation and % free-school meals within a school). Finally, we described service use within and across settings.

## Methods

### Study design and participants

We performed secondary data analysis on cross-sectional, pre-intervention data of the ‘My Resilience in Adolescence’ [MYRIAD] trial; a two-arm cluster (i.e., school-level) randomised controlled trial, aiming to improve adolescents' (N = 8376) mental health and well-being in 84 schools across the UK (ISRCTN86619085, 03/06/2016; see Appendix A).^20^ Data were collected across two cohorts, separated by 1 year. Each cohort provided pre-intervention data 1 year after the baseline assessment, following the randomisation of schools and before the delivery of the intervention to permit the training of teachers to deliver the intervention (see Fig. S1). The University of Oxford Central University Research Ethics Committee (R45358/RE001, 23/05/2016) provided ethical approvals for the MYRIAD trial. Other than a data access permission, no further approvals were required for this study. Participants gave informed consent to participate in the study before taking part. Informed consent/assent was collected from schools, parents (via opt-out of their child) and adolescents themselves. Schools were eligible if they were mainstream UK secondary schools (incl. private schools) that had a strategy and structure in place to deliver adequate social-emotional learning, a substantive appointed headteacher, and had not been rated ‘inadequate’ in their latest official school inspection. The trial sample was representative of UK secondary schools and adolescents.^21^

The complete set of study measures (incl. service use, pain, and suicidality) was first collected at pre-intervention (T1) in the academic years 2017/2018 (cohort 1: n = 923; 11.4%) and 2018/2019 (cohort 2: n = 7149; 88.6%) on 8072 adolescents (96.4% of trial participants) across 84 schools. Given our focus on pre-intervention data, we combined both trial arms into one group and controlled for trial arm in multivariable analyses. Additional information on the MYRIAD trial can be found in the trial protocol and update (Appendix A).^20^^,^^22^

### Measures

Additional details on study measures and references are provided in Supplement 1. Service use over 3 months was measured with the Child and Adolescent Service Use Schedule [CA-SUS]. Adolescents reported on use of hospital care, community health and social care services, medication for mental health problems (i.e., antidepressants, medication for sleep disorders and other (incl. attention deficit hyperactivity disorder, Tics/Tourette's, and psychosis)), looked-after care (foster/residential/respite care) and teaching support (Table S1). Each service item was multiplied by an appropriate unit cost for the financial year 2018–2019. Total costs per person were calculated by summing all costs within settings (total costs per setting) and across settings (total costs). This total service costs variable was used to obtain information on 1) whether adolescents have used services and hence had any associated service costs (i.e., service use vs no service use) and 2) average service costs in adolescents, who have used services.

The use of prescription pain medication was assessed with the item ‘*prescription painkillers/tranquilisers (e.g., Codeine or Valium)*’. No costs could be computed, as the quantity was not specified.

Persistent/recurrent pain over 6 months was measured using a composite binary pain variable that consisted of the pain item on the Strengths and Difficulties Questionnaire [SDQ]: In the past 6 months, ‘*I get a lot of headaches, stomach-aches, or sickness*’ and the Child Health Utility 9D [CHU-9D]: ‘*Are you in pain today?’*. We have used this composite ‘Pain’ variable to address the limitations of each single pain item and to capture potentially more persistent/recurrent (vs normative, acute) pain, which previous work has shown is more likely to be associated with future suicidality.^7^ Pain was coded ‘present’ if both items were endorsed (i.e., pain in the past 6 months (SDQ) and on the assessment day (CHU-9D)). If only one item (pain on the SDQ or CHU-9D) or neither pain item was endorsed, then pain was coded ‘absent’.

Suicidality was measured with three questions, inquiring about suicidal ideation and self-harm in the past 12 months; Adolescents were asked whether they had ‘(…) *thought that life was not worth living, or that [they] would be better off dead*’, ‘(…) *thought seriously about trying to harm [themselves] in some way*’ and ‘(…) *actually, deliberately harmed [themselves] in some way*’. Approximately 20% (n = 1611) of adolescents reported suicidality, including suicidal thoughts (n = 1280; 16%), self-harm thoughts (n = 898; 11%) and/or self-harm behaviours (n = 599; 7%). These low prevalence rates of suicidal and self-harm thoughts, as well as self-harm behaviours in this study rendered a separate exploration of thoughts and behaviours unfeasible. Hence, we combined all three questions into a binary ‘Suicidality’ variable. If at least one question was answered with ‘Yes’, suicidality was coded ‘present’. If all questions were answered with ‘No’ or ‘Prefer not to say’, suicidality was coded ‘absent’.

Individual characteristics included age (since last birthday), gender (self-identified boys/girls), and ethnicity (white/other). Additional individual characteristics were risk for depression (range = 0–60; Center for Epidemiologic Studies-Depression [CES-D]), anxiety symptoms (range = 0–114; Revised Children's Anxiety and Depression Scale [RCADS]) and peer problems over six months (range = 0–10; SDQ). Higher scores on each scale indicate greater difficulties.

School characteristics included school-area deprivation (Index of Multiple Deprivation [IMD 2015]; range = 1 ‘most deprived’ to 10 ‘least deprived’) and the proportion of students eligible for free school meals (economic deprivation; range 0%–100%).

### Statistical analysis

Statistical analyses were performed in R version 3.6.2^23^ (Table S2), using complete-case analyses, given the low proportions of missing data in the outcome (total service costs: 1%) and all school (0%) and individual characteristics (<3%), except anxiety symptoms and pain medication (6.0–9.2%). An exploration of missingness showed that adolescents with higher levels of anxiety symptoms were more likely to have missing cost data (see Table S3). Hence, finding effects in our more conservative, healthier retained sample would suggest that results are likely robust in the full sample. Furthermore, as the variable ‘pain medication’ was only used for descriptive purposes and not as an independent factor in the statistical analyses, we proceeded with complete-case analyses. We described prevalence rates of pain, suicidality, and pain-suicidality comorbidity. Participant characteristics were presented for the whole sample and separately for four subgroups (‘Group status’): adolescents with only suicidality (‘Suicidality’), only pain (‘Pain’), pain-suicidality comorbidity (‘Comorbidity’) and neither pain nor suicidality (‘Neither’). We plotted the proportion of adolescents using services by subgroup, setting, and service types, using bar plots. For each proportion, we added the respective two-sided 95% bootstrap confidence interval, using bias-corrected and accelerated bootstrapping (N = 10,000 samples), as an indication of precision and potential group differences. A one-way ANOVA was used to reveal overall group differences in total service costs. If group differences in total service costs were significant, then t-tests were used to compare total service costs in adolescents across groups. We corrected p-values for the false-discovery rate using Benjamini-Hochberg correction.

We estimated the intra-class correlation coefficient [ICC] for adolescents with any service costs to establish whether observations cluster within schools. As cost data were positively skewed (Fig. S2), we fitted two generalised linear mixed effects models, using a gamma distribution with log link, to estimate the ICCs and respective bootstrapped (N = 100) 95% confidence intervals. The first model refers to the unconditional means model (i.e., intercept only model). The second model refers to a conditional means model, including the fixed effect of group (‘Neither’ (Reference) vs ‘Suicidality’, ‘Pain’, and ‘Comorbidity’). As an additional indication of school-level clustering, we fitted two mixed-effect, random-intercept models with the fixed effect of group for a) students and b) students nested within schools. Effect estimates derived from both models were finally compared to those derived from a generalised linear model. Model selection was informed by a significant likelihood ratio test, goodness-of-fit criteria, and convergence warnings.

Owing to excess zeros and positively skewed cost data (Fig. S2), we estimated two-part hurdle models to test whether group status (‘Pain’, ‘Suicidality’ and ‘Comorbidity’ vs ‘Neither’ (reference)) is associated with service use and total service costs, and to explore the role of moderators (individual and school characteristics). The first part (service use vs no service use) revealed whether adolescents have used services and hence have had any associated costs. This part was described by a binominal distribution with logit link, which we inversed to obtain odds ratios [ORs]. The second part (positively skewed cost data) revealed the average total service costs in adolescents, who have used services. This part was described by a gamma distribution with log link, which we exponentiated to obtain incident rate ratios [IRRs]. Estimates from both parts were multiplied to obtain combined model estimates, showing the predicted costs downweighed by the probability of using services. As in community-based samples, the combined model estimates might hide important subgroup differences in either the use of services or the average total costs, we report the combined model estimates and describe both separate estimations (i.e., part 1 and 2) in detail. Univariable models were estimated to reveal possible pairwise associations and interactions with group status (factor-by-group interaction). We also estimated multivariable models, including group status, all individual and school characteristics, and significant factor-by-group interactions from univariable models. We controlled for cohort, trial arm, and multiple comparisons (Benjamini-Hochberg correction). All continuous factors were minimum centred to aid interpretation. Across analyses, we used bias-corrected and accelerated non-parametric bootstrapping (N = 10,000) to assess the stability of the results and associated level of certainty in the identified effects.^15^ Full details are reported in Supplement 2.

### Role of the funding source

The funders had no role in the study design, data collection, analysis, and interpretation, or writing of the manuscript. Authors had full access to all the data in the study and accept responsibility for the decision to submit for publication.

## Results

The study sample consisted of 8072 adolescents in 84 schools, with a low proportion of missing data for pain and suicidality (<1%), total service costs (1%), school (0%) and individual characteristics (anxiety symptoms/pain medication = 6.0–9.2%, all other characteristics<3%; Table 1 and Table S4). Proportions of missing data were higher when considering each service type (≤14%) and costs across settings (≤28%), as the response option ‘Don't know/don't want to say’ was coded as missing. Yet, service type and costs across settings were only used for descriptive purposes. Statistical analyses focussed on total service costs. Adolescents with (vs without) missing cost data were more often male and reported a higher risk of depression, anxiety symptoms, peer problems and use of prescription pain medication (Table S3).

**Table 1:** Participant characteristics at the pre-intervention assessment.

Variable	Subgroups^a^				Total (N = 8072)
	Neither (n = 5355; 66%)	Suicidality (n = 893; 11%)	Pain (n = 1074; 13%)	Comorbidity (n = 717; 9%)	
Cohort					
1, n (%)	635 (11.9)	84 (9.4)	144 (13.4)	58 (8.1)	923 (11.4)
2, n (%)	4720 (88.1)	809 (90.6)	930 (86.6)	**659 (91.9)**	7149 (88.6)
School characteristics (at the student-level)					
School-area deprivation (IMD; range: 1–10 (less deprivation); M (SD))	5.87 (2.71)	5.78 (2.66)	5.70 (2.72)	**5.93 (2.59)**	5.84 (2.69)
Percentage of students eligible for free-school meals (range = 0–100%); M (SD))	11.93 (8.86)	11.96 (8.71)	11.80 (8.95)	11.85 (8.45)	11.92 (8.84)
Adolescent demographics					
Age (range 11–15 years), M (SD)†	12.61 (0.61)	**12.70 (0.63)**	12.59 (0.60)	12.67 (0.62)	12.62 (0.61)
Gender					
Girls, n (%)	2655 (50.5)^q^	522 (59.4)^g^	683 (64.6)^b^	**510 (72.9)**^**b**^	4380 (55.3)^w^
Boys, n (%)	2518 (47.9)^q^	334 (38.0)^g^	353 (33.4)^b^	162 (23.1)^b^	3389 (42.8)^w^
Other/Prefer not to say, n (%)	86 (1.6)^q^	23 (2.6)^g^	21 (2.0)^b^	28 (4.0)^b^	158 (2.0)^w^
Ethnicity					
White, n (%)	3965 (75.7)^r^	646 (73.6)^h^	807 (76.6)^c^	528 (75.5)^n^	5967 (75.5)^x^
Asian, n (%)	537 (10.3)^r^	92 (10.5)^h^	119 (11.3)^c^	68 (9.7)^n^	819 (10.2)^x^
Black, n (%)	272 (5.2)^r^	52 (5.9)^h^	43 (4.1)^c^	37 (5.3)^n^	407 (5.2)^x^
Mixed and other ethnic minorities (e.g., Arab), n (%)	465 (8.9)^r^	88 (10.0)^h^	84 (8.0)^c^	66 (9.4)^n^	707 (8.9)^x^
Adolescents’ mental health and functioning					
Risk for depression (CES-D; range 0–60; M (SD))	11.32 (7.88)^s^	22.52 (10.23)^i^	19.95 (10.43)	**32.02 (10.75)**	15.58 (11.06)^n^
Low (0–15), n (%)	4025 (75.2)^s^	236 (26.5)^i^	424 (39.5)	44 (6.1)	4735 (58.8)^n^
At risk (16–27), n (%)	1095 (20.5)^s^	394 (44.2)^i^	395 (36.8)	208 (29.0)	2100 (26.1)^n^
Caseness (28–60), n (%)	232 (4.3)^s^	261 (29.3)^i^	255 (23.7)	**465 (64.9)**	1219 (15.1)^n^
Anxiety symptoms (RCADS; range 0–114; M (SD))	21.35 (15.31)^t^	38.40 (18.88)^j^	37.11 (19.43)^d^	**54.06 (20.78)**^**a**^	28.31 (19.98)^y^
RCADS T-score, M (SD)	42.67 (10.95)^u^	54.89 (13.51)^k^	53.89 (13.95)^e^	65.87 (14.81)^o^	47.57 (14.23)^z^
Non-clinical (T ≤ 64), n (%)	4684 (95.7)^u^	631 (78.2)^k^	776 (79.3)^e^	318 (49.4)^o^	6411 (87.5)^z^
Borderline (T: 65–69), n (%)	89 (1.8)^u^	67 (8.3)^k^	76 (7.8)^e^	81 (12.6)^o^	314 (4.3)^z^
Clinical (T ≥ 70), n (%)	120 (2.5)^u^	109 (13.5)^k^	127 (13.0)^e^	**245 (38.0)**^**o**^	602 (8.2)^z^
Peer problems (SDQ; range 0–10; M (SD))	1.64 (1.61)	2.80 (2.09)^l^	2.50 (1.93)	**3.61 (2.06)**	2.06 (1.88)^aa^
Normal (0–2), n (%)	4087 (76.3)^i^	457 (51.2)^l^	627 (58.4)	228 (31.8)	5401 (67.2)^aa^
Borderline (3), n (%)	596 (11.1)^i^	148 (16.6)^l^	173 (16.1)	140 (19.5)	1058 (13.2)^aa^
High (4), n (%)	336 (6.3)^i^	106 (11.9)^l^	99 (9.2)	122 (17.0)	663 (8.2)^aa^
Very high (5–10), n (%)	334 (6.2)^i^	181 (20.3)^l^	175 (16.3)	**227 (31.7)**	920 (11.4)^aa^
Pain medication, n (%)	99 (0.2)^v^	35 (4.3)^m^	42 (4.2)^f^	**49 (7.3)**^**p**^	226 (3.1)^ab^

Missing data: ^a.^*n* = 33, ^b.^*n* = 17, ^c.^*n* = 21, ^d.^*n* = 61, ^e.^*n* = 95, ^f.^*n* = 78, ^g.^*n* = 14, ^h.^*n* = 15, ^i.^*n* = 2, ^j.^*n* = 50, ^k.^*n* = 86, ^l.^*n* = 1, ^m.^*n* = 76, ^n.^*n* = 18, ^o.^*n* = 73, ^p.^*n* = 45, ^q.^*n* = 96, ^r.^*n* = 116, ^s.^*n* = 3, ^t.^*n* = 314, ^u.^*n* = 462, ^v.^*n* = 475, ^w.^*n* = 145, ^x.^*n* = 172, ^y.^*n* = 487, ^z.^*n* = 745, ^aa.^*n* = 30, ^ab.^*n* = 702. Total proportion of missing data per subgroup: Neither: anxiety/ pain medication = 5.9–8.9%, else <3%; Suicidality: anxiety/ pain medication = 5.6–9.6%, else <2%; Pain: anxiety/ pain medication = 5.7–8.8%, else <2%; Comorbidity: anxiety/ pain medication = 4.6–10.2%, else <3%; Total: anxiety/ pain medication = 6.0–9.2%, else <3%. Less than 1% had missing data on pain and/or suicidality. Legend: Neither = neither pain nor suicidality; Suicidality = suicidality but no pain; Pain = pain but no suicidality; Comorbidity = both pain and suicidality; Total = whole sample. The highest values across subgroups are printed in bold.

† Adolescents were mostly aged 12 (45%) or 13 (48%) years.

Participant characteristics are described in Table 1. Approximately 22% (n = 1794) of adolescents reported pain, 20% (n = 1611) suicidality, and 66% (n = 5355) neither pain nor suicidality. Pain was more common in adolescents with (vs without) suicidality and vice versa (Fig. S3), such that 9% (n = 717) of adolescents reported pain-suicidality comorbidity.

Compared to adolescents with neither pain nor suicidality, adolescents with pain and/or suicidality were more often female and reported a higher risk for depression, anxiety symptoms, peer problems, and more frequent use of prescription pain medication. These mean levels and proportions were highest for adolescents with pain-suicidality comorbidity compared to all other groups. Differences in both measures of school deprivation, age, or ethnicity were small between groups (Table 1).

### Total service use and associated costs

**Fig. 1:**
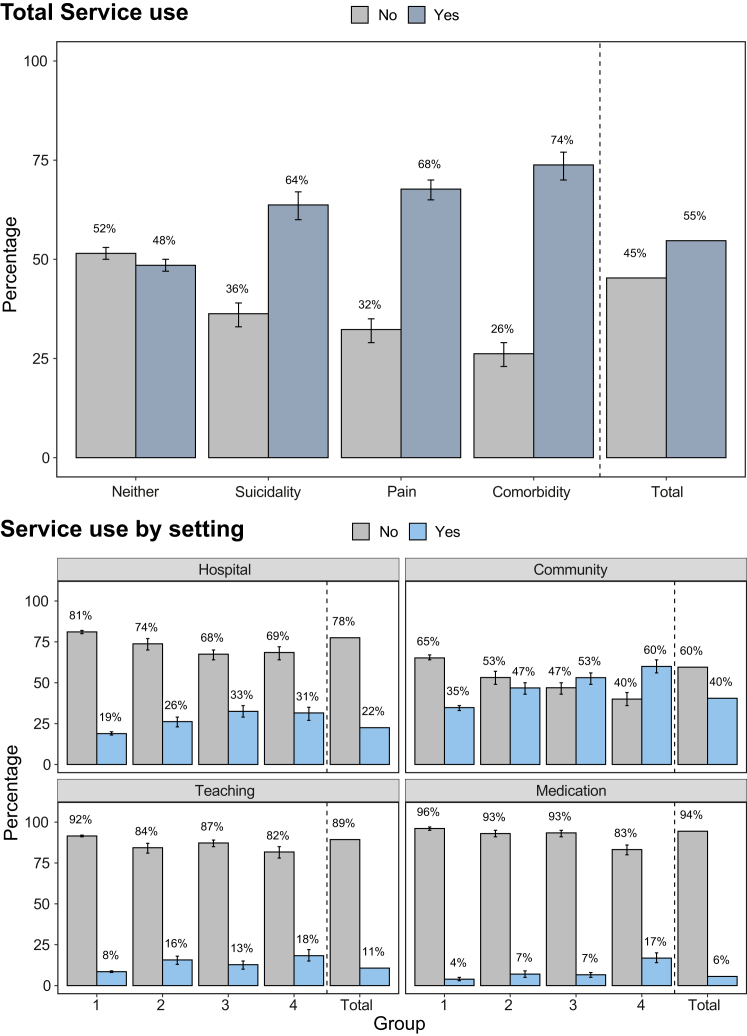
**Service use by subgroup and setting.** Group 1: Neither pain nor suicidality (n = 5355); Group 2: Suicidality without pain (n = 893); Group 3: Pain without suicidality (n = 1074); Group 4: Pain-suicidality comorbidity (n = 717); Total: N = 8072. Service use (no = £0; yes = at least £1) shows the proportion of adolescents using services across subgroups and settings. Looked-after care was excluded due to low numbers. Percentages, and their respective two-sided 95% bootstrap confidence intervals, using bias-corrected and accelerated bootstrapping (N = 10,000 samples), were computed and presented for those with available data (Tables S4 and S7)). A detailed breakdown of the number of adolescents with (vs without) service use in each group is provided in Table S6.

Overall, 55% of adolescents used services (Fig. 1). Service use was most common in adolescents with pain-suicidality comorbidity (74%), followed by adolescents with pain (68%), suicidality (64%), and neither pain nor suicidality (48%), with significant group differences in total service costs (p < 0.001; Table 2). Adolescents with pain and/or suicidality had significantly higher costs per person (Pain: £394; Suicidality: £392; Pain-Suicidality Comorbidity: £595), than adolescents with neither pain nor suicidality (£199). Total costs per person were highest for adolescents with pain-suicidality comorbidity, compared to adolescents with only pain or only suicidality (Table 2).

**Table 2:** Group differences in average service costs (£) per person.

Hypothesis 1: Higher total service costs in adolescents with only suicidality, only pain, and pain-suicidality comorbidity vs neither.					
Group	M (SD)	t	df	[95% CI]	p
Neither (reference)	£199 (456)				
Suicidality	£392 (686)	8.09	1012	[6.67–9.43]	**<0.001**
Pain	£394 (667)	9.11	1254	[7.69–10.52]	**<0.001**
Comorbidity	£595 (889)	11.51	734	[10.21–12.82]	**<0.001**

Hypothesis 2: Higher total service costs in adolescents with pain-suicidality comorbidity vs only suicidality or only pain.					
Group	M (SD)	t	df	[95%CI]	p
Comorbidity (reference)	£595 (889)				
Suicidality	£392 (686)	4.95	1261	[3.05–6.77]	**<0.001**
Pain	£394 (667)	5.07	1181	[3.19–6.79]	**<0.001**

Note. A one-way ANOVA revealed overall group differences (F(3) = 134.5, p < 0.001).

Neither: Adolescents, who reported neither pain nor suicidality; Suicidality = Adolescents, who reported suicidality but no pain; Pain = Adolescence, who reported pain but no suicidality; Comorbidity = Adolescents, who reported pain *and* suicidality. Confidence intervals were calculated across 10,000 bootstrap samples, using bias-corrected and accelerated non-parametric bootstrapping. p-values were corrected for the false-discovery rate using Benjamini-Hochberg correction. Significant group differences are printed in bold.

In a series of regression models, we explored the clustering of service costs in schools, and the role of individual and school characteristics. Given the low proportion of variance explained at the school-level (unconditional means model (without group status): ICC (95% CI) 1.4% (−0.5 to 3.3%); conditional means model (with group status): 0.7% (−0.9 to 2.5%)) and similar model estimates based on the two-level mixed effects and generalised linear models, we proceeded with generalised linear models, omitting school-level clustering, to reduce model complexity and aid model convergence (see Supplement 3).

Adjusted multivariable analyses (Table 3) showed that adolescents with pain (OR (95% CI) 3.79 (2.63–5.48)), suicidality (1.68 (1.12–2.51)), and pain-suicidality comorbidity (2.35 (1.26–4.41)) were more likely to use services, compared to adolescents with neither pain nor suicidality. If services were used, then adolescents with pain and/or suicidality were more likely to have higher costs (Pain: IRR (95% CI) 1.25 (1.11–1.42); Suicidality: 1.27 (1.11–1.46); Comorbidity: 1.57 (1.34–1.85)), compared to adolescents with neither pain nor suicidality. These findings are consistent with univariable analyses (Table S5).

**Table 3:** Multivariable analyses (adjusted for cohort, trial arm, & multiple comparisons).

Factors	Service use: Odds ratio (OR) (binominal component)			Service costs if services were used: Incident rate ratios (IRR), (gamma component)			Combination of both models	
	Model-based estimates			Model-based estimates			Bootstrapped estimates	
	OR	95% CI	p	IRR	95% CI	p	IRR	95% CI
**Multivariable model**								
Group: Suicidality	1.68	[1.12, 2.51]	**0.011**^a^	1.27	[1.11, 1.46]	**<0.001**^a^	0.79	[0.65, 0.96]
Group: Pain	3.79	[2.63, 5.48]	**<0.001**^a^	1.25	[1.11, 1.42]	**<0.001**^a^	0.99	[0.86, 1.13]
Group: Comorbidity	2.35	[1.26, 4.41]	**0.007**^a^	1.57	[1.34, 1.85]	**<0.001**^a^	1.10	[0.84, 1.37]
Age^b^	1.04	[0.96, 1.12]	0.338	1.05	[0.99, 1.13]	0.128	0.54	[0.50, 0.58]
Group(Suicidality)∗Age^b^	NA	NA	NA	NA	NA	NA	NA	NA
Group(Pain)∗Age^b^	NA	NA	NA	NA	NA	NA	NA	NA
Group(Comorbidity)∗Age^b^	NA	NA	NA	NA	NA	NA	NA	NA
Gender(Female)	1.06	[0.94, 1.20]	0.321	0.81	[0.74, 0.88]	**<0.001**^a^	0.41	[0.37, 0.46]
Group(Suicidality)∗Gender(F)	1.12	[0.80, 1.56]	0.517	NA	NA	NA	0.42	[0.35, 0.51]
Group(Pain)∗Gender(F)	0.81	[0.58, 1.11]	0.189	NA	NA	NA	0.36	[0.29, 0.43]
Group(Comorbidity)∗Gender(F)	1.26	[0.81, 1.95]	0.295	NA	NA	NA	0.45	[0.35, 0.55]
Ethnicity(White)	0.91	[0.82, 1.02]	0.123	1.04	[0.94, 1.14]	0.450	0.50	[0.44, 0.56]
Group(Suicidality)∗Ethnicity(W)	NA	NA	NA	NA	NA	NA	NA	NA
Group(Pain)∗Ethnicity(W)	NA	NA	NA	NA	NA	NA	NA	NA
Group(Comorbidity)∗Ethnicity(W)	NA	NA	NA	NA	NA	NA	NA	NA
Risk for depression^b^	1.01	[1.00, 1.02]	**0.005**^a^	1.01	[1.00, 1.02]	**<0.001**^a^	0.51	[0.50, 0.51]
Group(Suicidality)∗Depression^b^	1.01	[0.99, 1.03]	0.404	NA	NA	NA	0.51	[0.50, 0.51]
Group(Pain)∗Depression^b^	0.98	[0.97, 1.00]	0.088	NA	NA	NA	0.50	[0.50, 0.51]
Group(Comorbidity)∗Depression^b^	0.99	[0.97, 1.01]	0.404	NA	NA	NA	0.50	[0.50, 0.51]
Anxiety symptoms^b^	1.01	[1.01, 1.02]	**<0.001**^a^	1.00	[1.00, 1.01]	**0.027**^a^	0.51	[0.50, 0.51]
Group(Suicidality)∗Anxiety^b^	0.99	[0.98, 1.00]	**0.016**^a^	NA	NA	NA	0.50	[0.49, 0.50]
Group(Pain)∗Anxiety^b^	0.99	[0.98, 1.00]	**0.039**^a^	NA	NA	NA	0.50	[0.50, 0.50]
Group(Comorbidity)∗Anxiety^b^	0.99	[0.98, 1.01]	0.280	NA	NA	NA	0.50	[0.50, 0.50]
Peer problems^b^	1.01	[0.98, 1.04]	0.630	1.01	[0.99, 1.04]	0.391	0.51	[0.49, 0.52]
Group(Suicidality)∗Peer^b^	NA	NA	NA	NA	NA	NA	NA	NA
Group(Pain)∗Peer^b^	NA	NA	NA	NA	NA	NA	NA	NA
Group(Comorbidity)∗Peer^b^	NA	NA	NA	NA	NA	NA	NA	NA
School-area deprivation^b^	1.02	[1.00, 1.04]	0.082	0.99	[0.97, 1.01]	0.220	0.50	[0.49, 0.51]
Group(Suicidality)∗Deprivation^b^	NA	NA	NA	NA	NA	NA	NA	NA
Group(Pain)∗Deprivation^b^	NA	NA	NA	NA	NA	NA	NA	NA
Group(Comorbidity)∗Deprivation^b^	NA	NA	NA	NA	NA	NA	NA	NA
Free-school meals^b^	1.00	[0.99, 1.00]	0.390	1.01	[1.00, 1.01]	**0.020**^a^	0.50	[0.50, 0.51]
Group(Suicidality)∗Meals^b^	NA	NA	NA	NA	NA	NA	NA	NA
Group(Pain)∗Meals^b^	NA	NA	NA	NA	NA	NA	NA	NA
Group(Comorbidity)∗Meals^b^	NA	NA	NA	NA	NA	NA	NA	NA

Note. Suicidality = Adolescents, who reported suicidality but no pain; Pain = Adolescence, who reported pain but no suicidality; Comorbidity = Adolescents, who reported pain *and* suicidality; Reference group: Adolescents, who reported neither pain nor suicidality. NA = only the main association, but not the factor by group status interaction term, was included in the model.

a Significant (p < 0.05), following adjustment for multiple comparisons (BH adjustment).

b Minimum centred.

Across groups, adolescents with a higher risk for depression and anxiety symptoms were more likely to use services (Table 3). Yet, some adolescents with suicidality (36%), pain (32%) and pain-suicidality comorbidity (26%) did not use services, despite experiencing depression and anxiety symptoms in addition to their pain and/or suicidality (Table S6). If services were used, male gender, higher risk for depression, anxiety symptoms and percentage of free-school meals were associated with a higher likelihood of higher service costs (Table 3). Additional associations were revealed in univariable analyses (Table S5), which diminished in adjusted, multivariable analyses (Table 3).

### Service use and costs by settings

Compared to adolescents with neither pain nor suicidality, proportionally more adolescents with pain-suicidality comorbidity used hospital, community, and school services, as well as medication for mental health difficulties (see the 95% confidence intervals in Fig. 1 and Table S7) and had higher service costs (Table S4). Furthermore, proportionally more adolescents with pain-suicidality comorbidity used i) community services compared to adolescents with only suicidality and ii) medication for mental health difficulties compared to adolescents with only suicidality or only pain (see the 95% confidence intervals in Fig. 1 and Table S7).

**Fig. 2:**
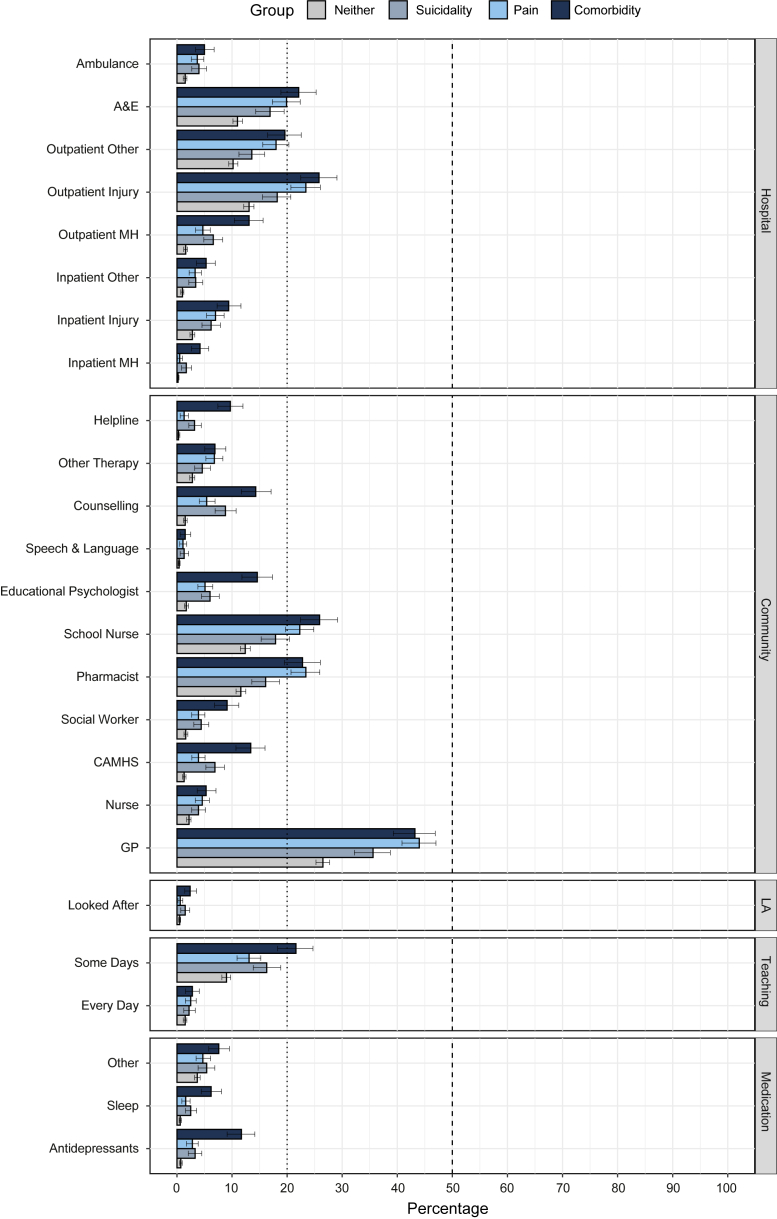
**Service use by subgroup, setting and service type.** A detailed breakdown of each service setting is provided, showing the service items that adolescents selected. Neither = neither pain nor suicidality; Suicidality = suicidality but no pain; Pain = pain but no suicidality; Comorbidity = both pain and suicidality; MH = Mental health, A&E = Accident & Emergency, CAMHS = Child & Adolescent Mental Health Services, LA = Looked-after care (incl. foster care, residential care, and respite care), Teaching = Teaching Support, Medication (‘other’): medication for tics/Tourette's, Attention Deficit Hyperactivity Disorder [ADHD], Psychosis. Percentages, and their respective two-sided 95% bootstrap confidence intervals, using bias-corrected and accelerated bootstrapping (N = 10,000 samples), were computed and presented for those with available data (Tables S7 and S8).

Across subgroups, the following service types were most frequently reported: general practitioner [GP] visits, outpatient appointments for injuries, and contacts with a school nurse or pharmacist (Fig. 2 and Table S8). Although GP visits were most common, more than half of adolescents across subgroups have not been in contact with their GP. The use of hospital and community services related to mental health difficulties was low across subgroups, but highest for adolescents with pain-suicidality comorbidity (i.e., hospital: inpatient and outpatient mental health services; community: CAMHS, social worker, education psychologist, counselling, and helplines; see the 95% confidence intervals in Fig. 2, Tables S7 and S8). Across subgroups, the use of medication for mental health problems was low, but highest for adolescents with pain-suicidality comorbidity, especially for antidepressants and sleep medication (see the 95% confidence intervals in Fig. 2, Tables S7 and S8).

## Discussion

We explored service use and total service costs over three months in adolescents with persistent/recurrent pain and/or suicidality in the UK, and the role of individual and school characteristics. Key findings include: 1) substantial comorbidity between pain and suicidality in adolescence, 2) one-in-two adolescents reported service use, especially GP visits, outpatient appointments for injuries and contacts with a school nurse or pharmacist, and 3) adolescents with pain and/or suicidality were more likely than adolescents with neither pain nor suicidality to use services and to have higher total costs. The increased contact with services in adolescents with pain may provide an opportunity for suicide prevention. Findings underscore the need for multi-sector suicide prevention strategies in hospital, community, and school settings.

Prevalence rates of pain and suicidality in adolescents are well-established^2^^,^^9^ and align with observed prevalence rates of pain (22%) or suicidality (20%). Our findings add to this literature by quantifying the pain-suicidality comorbidity in adolescence (9%). Compared to all other groups, adolescents with pain-suicidality comorbidity reported higher levels of depression, anxiety symptoms, peer problems, and use of prescription pain medication. Indeed, mental health and peer problems are key correlates of pain and suicidality in adolescence.^16^^,^^24^ Finding the highest levels of these difficulties in adolescents with pain-suicidality comorbidity emphasises the combined burden, which together with an increased access to potentially lethal means (pain medication) might increase suicidal risk in some adolescents. Adolescents with pain and/or suicidality were more often female, which is well-established for pain or suicidality.^1^^,^^2^^,^^10^ Yet, much less is known about the role of gender in the context of pain-suicidality comorbidity, which awaits future research.

Approximately half the adolescents used services over 3 months. As hypothesised and consistent with the literature in adults,^18^^,^^19^ total service use and associated costs were highest for adolescents with pain-suicidality comorbidity compared to all other groups. Adolescents with pain and/or suicidality (vs neither) were 2–4 times more likely to use services and to have higher total costs, especially adolescents with pain-suicidality comorbidity. Higher rates of service use in the presence of physical and mental health comorbidities might be explained by the combined burden, increased service contacts for physical problems that may facilitate direct referrals and treatment continuation, or the perception of physical risk related to mental health problems.^18^ Therefore, clinicians should be alert to the risk of self-harm and suicidal ideation in this group.

However, a relatively large proportion of adolescents did not use services even when experiencing pain and/or suicidality (26–36%) or used few services associated with only minimal costs, possibly due to limited access to more costly services or to less frequent usage. This finding aligns with the adolescent pain literature, showing that 66% of all service costs were explained by the top 5% of adolescents with the highest costs^13^ and suggest the need for service optimisations, targeted support, and access to evidence-based information about coping with psychological distress and physical pain.

Across groups, higher levels of depression and anxiety symptoms were associated with a higher likelihood of service use and higher total costs. Yet, the lack of interactions with group status, suggests that some adolescents do not use services despite experiencing depression and anxiety symptoms in addition to their pain and/or suicidality. Therefore, improved screening and tailored care is vital to reach all vulnerable youth. Similarly, if costs were incurred, boys had higher service costs across groups, which suggests that boys and girls may use or be offered different types of services, which awaits further research. The finding that adolescents in schools with a higher proportion of free-school meals were more likely to have higher service costs, might show how universal healthcare access in the UK^25^ can facilitate adolescents’ contact with professional services, compared to alternative healthcare models where healthcare access is dependent on medical insurance status and associated with higher income.^26^

The pattern of service use was similar across groups. Although GP visits were most frequently reported, most adolescents had not been in contact with their GP. Despite the key role of GP services in the identification, management, referral, and ongoing support for suicidal risk in adolescents in the community,^27^ these findings suggest that sole reliance on GP services may not be sufficient. Given the diversity of identified services, it is crucial to increase awareness of suicidal risk in adolescents with pain across settings to aid early risk identification, timely referrals, and tailored support. Use of services related to mental health problems was low across subgroups, but highest for adolescents with pain-suicidality comorbidity. Explanations for low service use may include the young persons' perception of being able or ought to be able to self-manage one's mental health difficulties, perceived lack of importance, seriousness or mental health knowledge, perceived stigma, or lack of trust in the therapeutic relationship, and perceived structural barriers (logistics, lack of service access or family support),^17^^,^^28^ which requires further research to inform support strategies.

Finally, the use of medication for mental health problems was typically low, but highest for adolescents with pain-suicidality comorbidity. As access to potentially lethal means might enhance suicidal capacity,^29^ safety assessments and ongoing risk monitoring (including monitoring of prescribed medication load) in adolescents with pain and/or suicidality is paramount, whilst active treatment for both pain and mental health conditions remains important to reduce distress and improve functioning.

This study has limitations: First, pain items were not exclusively pain-specific, and characteristics of the pain experience remain unknown. Yet, the combination of both items into a composite measure addresses the limitations of each separate pain item and makes the presence of persistent/recurrent pain more likely. Second, consistent with population-based research,^9^ self-harm behaviours, irrespective of suicidal intent, were only endorsed by a small proportion (7%) of the study sample, rending a separate exploration of suicidal thoughts and behaviours unfeasible. Hence, we created a composite ‘Suicidality’ variable, capturing both self-harm thoughts and behaviours, irrespective of suicidal intent, which aligns with the view that suicidal intent is a dimensional phenomenon.^16^ Third, although we selected a broad range of individual and school characteristics, the use of secondary data meant that we were constrained by the measures available. To develop a more complete understanding of the role of other unmeasured individual or school characteristics that could explain service use and costs in adolescents with pain and/or suicidality, future research should explore a broader range of factors. Fourth, the reasons for adolescents' service use were not assessed, leaving it unclear whether adolescents have received support for pain and/or suicidality, for other mental health difficulties (co-occurring depressive or anxiety symptoms) or for an unrelated problem. Previous research has shown that most adolescents with suicidality (55–73%) have been in contact with professional services 12 months prior to their experience of suicidality.^30^ Thus, even if adolescents use services, suicidality is not always successfully prevented.^30^ Future research should explore the reasons for and effectiveness of service use in adolescents with pain and/or suicidality to optimise services. Fifth, informal support (e.g., friends or family)^10^ was not assessed and its use remains unknown. Sixth, no conclusions can be drawn about the causal direction of these cross-sectional associations. Future research should establish whether service use increases because of e.g., the combined burden associated with pain-suicidality comorbidity^18^ or whether suicidality may develop because of e.g., unsuccessful pain management.

Findings emphasise the common comorbidity between pain and suicidality in adolescence. About one-in-two adolescents reported service use, with a higher proportion of adolescents with pain-suicidality comorbidity using services related to mental health difficulties. Yet, given the diversity of identified settings sole reliance on primary care (GPs) to assess and address suicidal risk in adolescence is not sufficient. A multi-sector approach to suicidal risk assessment and management is required to address suicidality in the community. This includes training in pain management and suicidal risk identification across settings, appropriate referrals where necessary, and addressing barriers of specialised mental health services use. Importantly, the presence of pain may offer an opportunity for service providers to screen for suicidal risk and offer timely support if needed.

## Contributors

V.H. conceived the study idea. V.H. developed the study proposal with input from all authors. S.B. was responsible for the economic component of the trial, including the service use measure and the cost data. P.G. performed the cost calculations for the trial. V.H. performed all analyses reported in the manuscript, with advice from J.M.-M. and P.G. V.H., J.M.-M. and P.G. have verified the underlying data. V.H. drafted the manuscript. All authors read, commented on, and approved the final manuscript. Authors had full access to all the data in the study and accept responsibility for the decision to submit for publication.

## Data sharing statement

Data are available upon reasonable request. The de-identified data and codebook from the MYRIAD trial are available from Prof. Kuyken (willem.kuyken@psych.ox.ac.uk) upon request. Release of data is subject to an approved proposal and a signed data access agreement. Syntax files related to this study will be released on the Open Science Framework upon publication (project title: ‘*Shedding Light on Service Use and Costs in Adolescents with Pain and Suicidality*’; link: https://osf.io/ckx23/?view_only=cfd1e15197fe4b828c19792a963ccd14).

## Declaration of interests

We declare no competing interests.
